# Supplementary material for: Maturation and specialization of group 2 innate lymphoid cells through the lung-gut axis
Source: Nat Commun. 2022 Dec 9;13:7600. doi: 10.1038/s41467-022-35347-6 (PMC9734379; doi:10.1038/s41467-022-35347-6)
Supplement: Supplementary file 1 — Supplementary Information [file 41467_2022_35347_MOESM1_ESM.pdf]

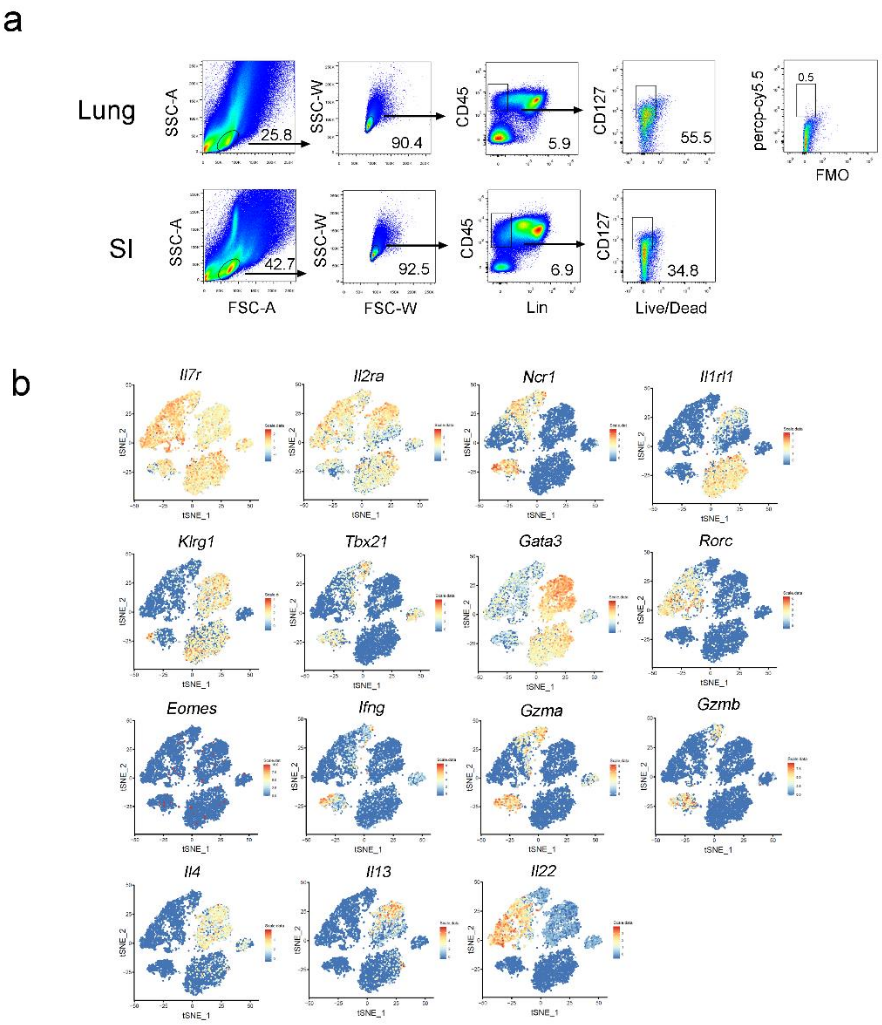

Supplementary Fig. 1. Single cell RNA sequencing of ILCs in the small intestine and lung. a, Gating strategies for ILC isolation by flow cytometry. ILCs were isolated from the small intestine (SI) and lung from wild type mice and sorted by flow cytometry. Fluorescence Minus One (FMO) showed as gate staining control of CD127. (Lin=CD3e,CD8a,CD19,CD11b,CD11c,Gr1,F4/80,Ter119). **b,** Displaying of normalized expression levels of ILC signature genes on a tSNE plot from Fig. 1b.

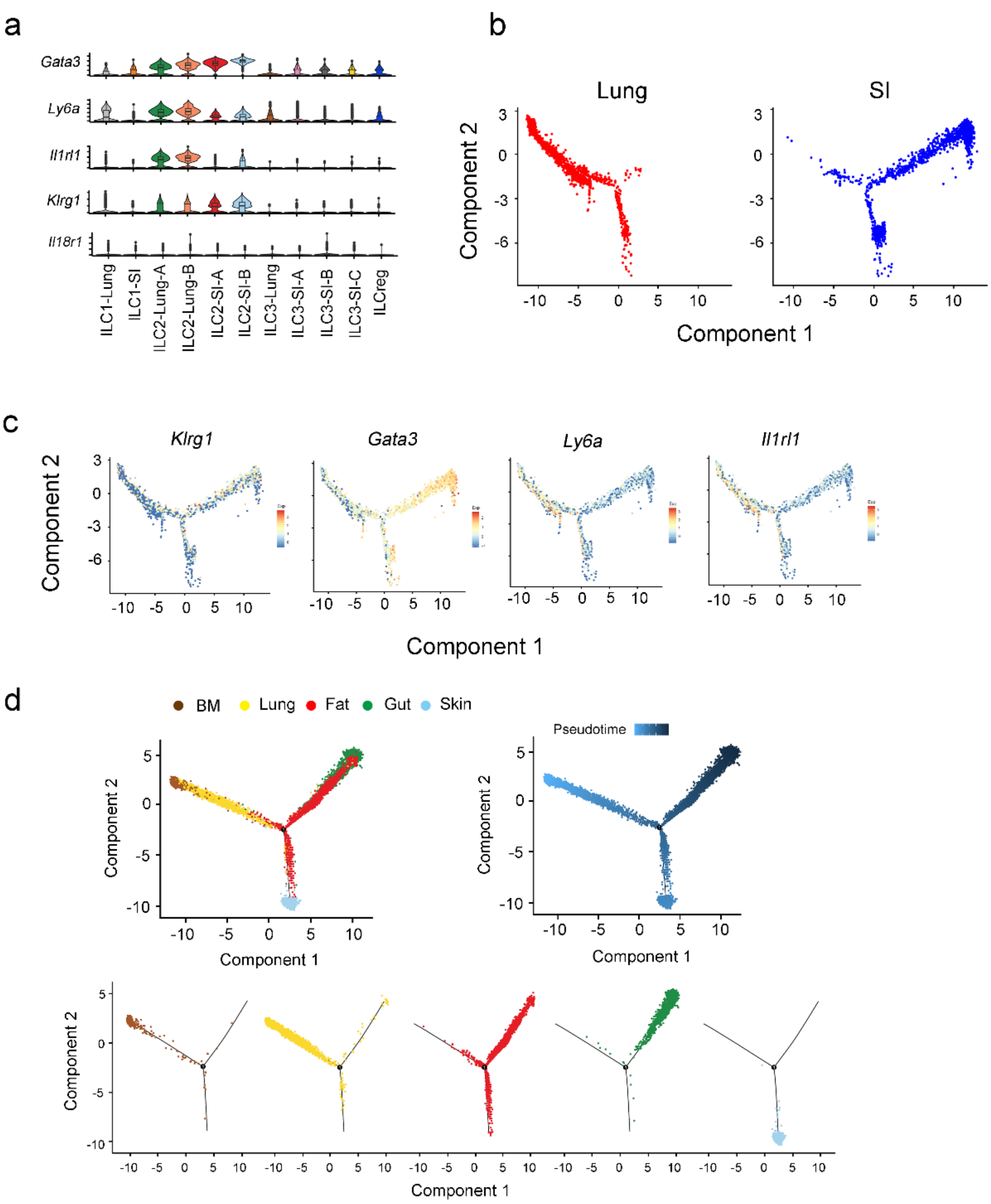

Supplementary Fig. 2. Analysis of ILC2 signature genes and maturation trajectory. a, Violin plots displaying ILC2-related genes in each cell cluster. Box plots indicate median (middle line), 25th, 75th percentile (box) of the data and the maximum and minimum values as endpoints for the whiskers. **b,** Distribution of lung and intestine ILC2s on pseudotime plot. **c,** Normalized expression levels of selected genes overlaid on Monocle2 pseudotime plot. **d,** Pseudotime plot of ILC2 subsets from the indicated tissues according to scRNA-sequencing data from previous report [22].

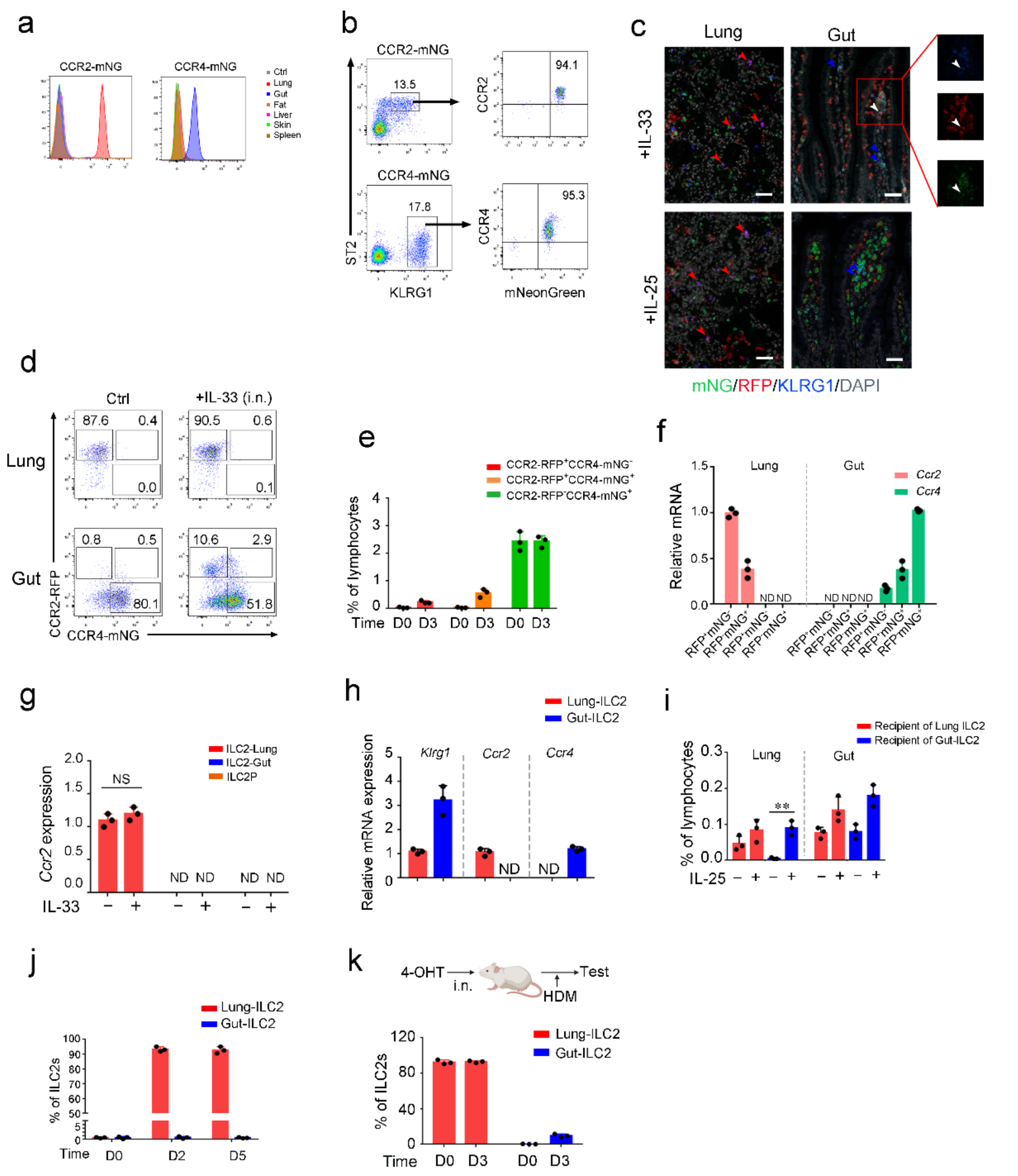

Supplementary Fig. 3. Maturation of ILC2s via lung-gut axis. a, Expression of CCR2-mNeonGreen and CCR4-mNeonGreen on ILC2s from indicated tissues of CCR2-mNeonGreen reporter mice and CCR4-mNeonGreen reporter mice were analyzed by flow cytometry. WT mice served as a negative control. **b,** Expression levels of CCR2 on lung ILC2s or CCR4 on gut ILC2s from CCR2-mNeonGreen or CCR2-mNeonGreen reporter mice respectively was analyzed by flow cytometry. **c,** CCR2-RFP;CCR4-mNeonGreen mice were i.p. injected with IL-25 or IL-33. After 72 hours, the lung or gut tissues of mice were collected and fixed. The sections of lung and gut tissues from the treated mice were subjected to the immunostaining of indicated antibodies. Red arrowhead, RFP^{+}ILC2; Blue arrowhead, mNG^{+}ILC2; White arrowhead, RFP^{+}mNG^{+}ILC2. Scale bar, 100 μm. Data are representative of two independent experiments. **d,** CCR2-RFP;CCR4-mNeonGreen (mNG) mice were intranasally treated with IL-33 (200 ng/mouse) for 1 day and ILC2s from lung and

gut were analyzed by flow cytometry. **e,** Analysis of mNeonGreen and RPF expression of gut ILC2s from CCR2-RFP;CCR4-mNeonGreen mice after intranasal administration of 10 μg HDM/mouse for the indicated days. The cell frequency of indicated subgroups of gut ILC2s was calculated and shown as means±SD. (n=3 for each group). **f,** mRNA expression levels of *Ccr2* and *Ccr4* genes in the indicated ILC2 subsets from CCR2-RFP;CCR4-mNeonGreen (mNG) mice with i.p. injection of IL-33 was analyzed by real-time (RT)-PCR and shown as means±SD. mNG, mNeonGreen. ND, not detectable. **g,** ILC2s were isolated from the lung and the gut, and ILC2Ps were isolated from BM. ILC2s and ILC2Ps were treated with 10ng/ml IL-33 for 24 h in vitro. The expression levels of *Ccr2* were analyzed by qPCR assay. The relative mRNA levels were shown as means±SD. ND, not detectable. NS, not significant (P>0.05) by Two-tailed unpaired Student's t-test. **h-i,** Adoptive transfer of lung or gut ILC2s ($5x10^4$) from WT CD45.1 mice to B-NDG mice. Six weeks after of transfer, the ILC2s from lung-ILC2 transferred mice were harvested for qPCR assay of indicated genes. The relative expression of the indicated genes was shown as means±SD. **, P<0.01 by Two-tailed unpaired Student's t-test. (P=0.0019) (h). Alternatively, the recipient mice were treated with or without 200 ng/mouse IL-25 i.p. for one day. The lung and gut ILC2s from the recipient mice were analyzed by flow cytometry. The cell frequency of ILC2s were calculated and shown as means±SD (i). **j,** Solubilizing 4-OHT (2 μg/g mouse) were atomized and delivered into lung of *Id2-Cre/ERT2;Rosa26-STOP-tdTomato* mice by liquid aerosol devices. After indicated days of 4-OHT treatment, lung and gut ILC2s were analyzed by flow cytometry and the frequency of TdTomato$^+$ ILC2s were calculated and show as means±SD. **k,** Solubilizing 4-OHT (2 μg/g mouse) were atomized and delivered into lung of *Id2-Cre/ERT2;Rosa26-STOP-TdTomato* mice by liquid aerosol devices. Two days after 4-OHT treatment, these mice were intranasal administration of 10 μg HDM/mouse for the indicated days and the frequency of TdTomato$^+$ ILC2s were calculated and show as means±SD. The schematic diagram was created with BioRender.com.

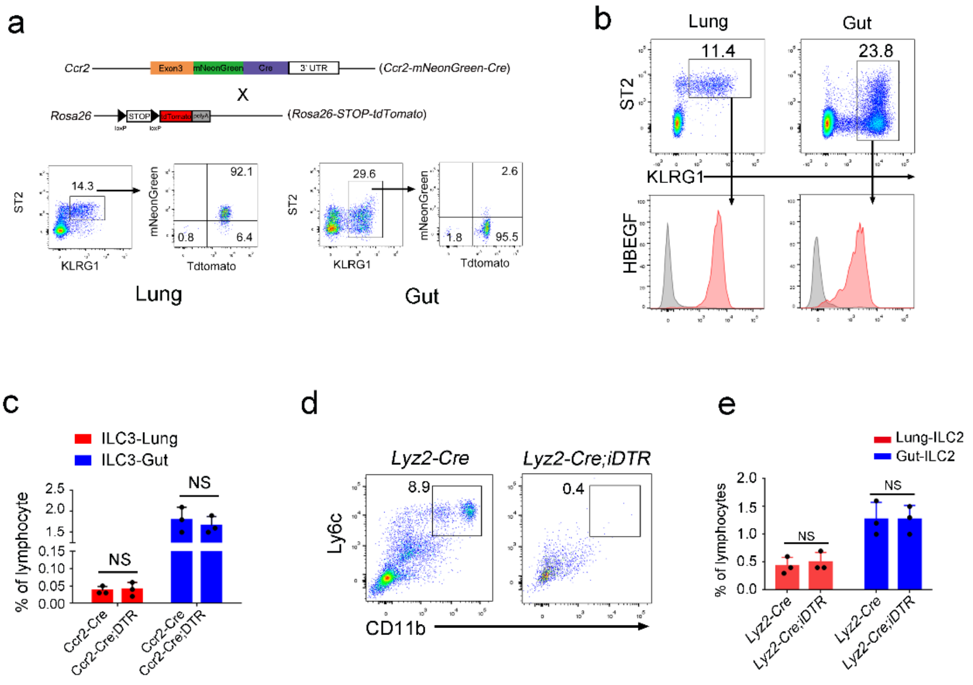

Supplementary Fig. 4. Depletion and tracing of ILC2s in the lung and gut tissues. a, Lineage tracing of CCR2^{+}ILC2s. Lung or gut ILC2s from *Ccr2-mNeonGreen-Cre;Rosa26-STOP-tdTomato* mice were analyzed by flow cytometry. **b,** Expression of diphtheria toxin receptor (HBEGF) on ILC2s. The lung and gut ILC2s from *CCR2-mNeonGreen-Cre;Rosa26-STOP-DTR* mice were stained with antibodies against ILC2 markers and DTR (HBEGF) and analyzed by flow cytometry. **c,** Analysis of ILC3s in ILC2 depletion mice. *Ccr2-mNeonGreen-Cre;Rosa26-STOP-DTR* mice were subjected to i.p. injection of 100 ng/mouse diphtheria (DT) every two days. Cell frequency of ILC3s from the lung and gut were analyzed by flow cytometry and shown as means±SD. NS, not significant (P>0.05) by Two-tailed unpaired Student's t-test. (n=3 for each group). **d-e,** Depletion of myeloid cells. *Lyz2-Cre;Rosa26-STOP-DTR* mice were subjected to i.p. injection of 100 ng/mouse diphtheria (DT) every two days for six days. Cell frequency of monocytes from the lung were analyzed by flow cytometry (d) and the cell frequency of ILC2s were shown as means±SD (e). (n=3 for each group). NS, not significant (P>0.05) by Two-tailed unpaired Student's t-test.

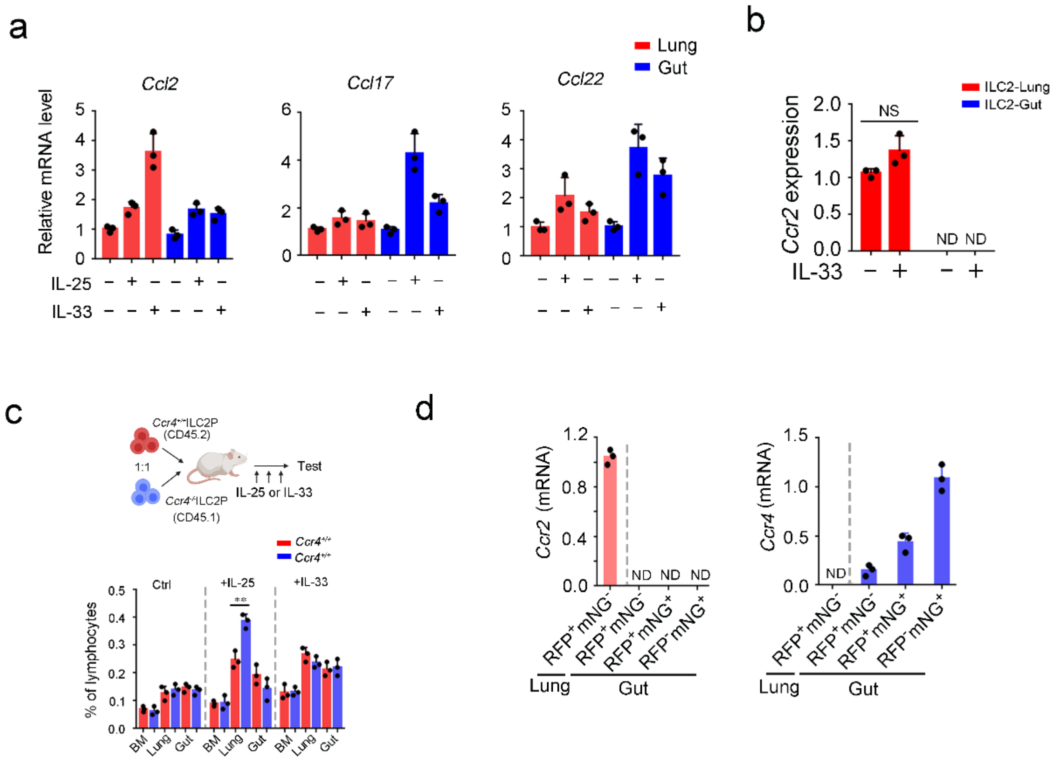

Supplementary Fig. 5. CCR2 and CCR4 signaling regulate ILC2 translocation. a, The expression levels of *Ccl2* (CCR2 ligand) and *Ccl17*/*Ccl22* (CCR4 ligand) were analyzed by qPCR assay. Epithelial cells from the lung and gut were isolated from the mice i.p. treated with 400 ng/mouse IL-33 or 200 ng/mouse IL-25 for 1 day and subjected to mRNA extraction for qPCR. The relative mRNA levels were shown as means±SD. **b,** The expression levels of *Ccr2* were analyzed by qPCR assay. The lung and gut ILC2s were isolated from WT mice i.p. treated with IL-33 for 1 day and subjected to mRNA extraction for qPCR. The relative mRNA levels were and shown as means±SD. ND, not detectable. NS, not significant (P>0.05) by Two-tailed unpaired Student's *t*-test. **c,** Adoptive transfer of WT and $Ccr4^{-/-}$ ILC2Ps. ILC2Ps ($Lin^{-}CD127^{+}Sca1^{+}ST2^{+}KLRG1^{-}$) were isolated from BM of WT and $Ccr4^{-/-}$ mice. WT and $Ccr4^{-/-}$ ILC2Ps were 1:1 mixed ($5x10^{4}$ for each) and i.v. injected into B-NDG mice. One week after transfer, the recipient mice were i.p. injected with 400 ng/mouse/day IL-33 or 200 ng/mouse/day IL-25 for three days. The cell frequency of ILC2s in the lung and gut were calculated and shown as means±SD. **, P<0.01 by Two-tailed unpaired Student's *t*-test (P=0.0036). (n=3 for each group). The schematic diagram was created with BioRender.com. **d,** mRNA levels of *Ccr2* and *Ccr4* in ILC2s from CCR2-RFP;CCR4-mNeonGreen mice at postnatal day 12 was analyzed by qPCR assay. The relative expression levels of *Ccr2* and *Ccr4* from indicated subsets were analyzed and shown as means±SD. ND, not detectable. (n=3 for each group). Data are representative of three independent experiments.
