# Supplementary material for: Maturation and specialization of group 2 innate lymphoid cells through the lung-gut axis
Source: Nat Commun. 2022 Dec 9;13:7600. doi: 10.1038/s41467-022-35347-6 (PMC9734379; doi:10.1038/s41467-022-35347-6)
Supplement: Supplementary file 3 — Description of Additional Supplementary Files [file 41467_2022_35347_MOESM3_ESM.pdf]

Description of Additional Supplementary Files

File Name: Supplementary Data 1

Description:

Three data sheets were included, i.e sheet ILC1, sheet ILC2, sheet ILC3.

Sheet ILC1, GO items and the respective gene list of Figure 2d. Genes of ILC1 that up-regulated in lung and small intestine were used for analysis.

Sheet ILC2. GO items and the respective gene list of Figure 2e. Genes of ILC2 that up-regulated in lung and small intestine were used for analysis.

Sheet ILC3. GO items and the respective gene list of Figure 2f. Genes of ILC3 that up-regulated in lung and small intestine were used for analysis.
