# Supplementary material for: Maturation and specialization of group 2 innate lymphoid cells through the lung-gut axis
Source: Nat Commun. 2022 Dec 9;13:7600. doi: 10.1038/s41467-022-35347-6 (PMC9734379; doi:10.1038/s41467-022-35347-6)
Supplement: Supplementary file 5 — Reporting Summary [file 41467_2022_35347_MOESM5_ESM.pdf]

nature portfolio

Corresponding author(s): Shuo Wang

Last updated by author(s): Nov 14, 2022

Reporting Summary

Nature Portfolio wishes to improve the reproducibility of the work that we publish. This form provides structure for consistency and transparency in reporting. For further information on Nature Portfolio policies, see our Editorial Policies and the Editorial Policy Checklist.

Statistics

For all statistical analyses, confirm that the following items are present in the figure legend, table legend, main text, or Methods section.

n/a	Confirmed	
☐	☒	The exact sample size (*n*) for each experimental group/condition, given as a discrete number and unit of measurement
☐	☒	A statement on whether measurements were taken from distinct samples or whether the same sample was measured repeatedly
☐	☒	The statistical test(s) used AND whether they are one- or two-sided<br>*Only common tests should be described solely by name; describe more complex techniques in the Methods section.*
☐	☒	A description of all covariates tested
☐	☒	A description of any assumptions or corrections, such as tests of normality and adjustment for multiple comparisons
☐	☒	A full description of the statistical parameters including central tendency (e.g. means) or other basic estimates (e.g. regression coefficient) AND variation (e.g. standard deviation) or associated estimates of uncertainty (e.g. confidence intervals)
☐	☒	For null hypothesis testing, the test statistic (e.g. F, t, r) with confidence intervals, effect sizes, degrees of freedom and P value noted<br>*Give P values as exact values whenever suitable.*
☐	☒	For Bayesian analysis, information on the choice of priors and Markov chain Monte Carlo settings
☐	☒	For hierarchical and complex designs, identification of the appropriate level for tests and full reporting of outcomes
☐	☒	Estimates of effect sizes (e.g. Cohen's d, Pearson's r), indicating how they were calculated

Our web collection on statistics for biologists contains articles on many of the points above.

Software and code

Policy information about availability of computer code

Data collection	For scRNA-sequencing, we aligned reads to the mouse mm10 genome by using CellRanger software (version 3.1.5) to generate the single cell information.
Data analysis	For scRNA-sequencing, we excluded doublets, poor-quality cells and contamination cells by Seurat software (version 3.1.5) . We used scRNA-seq data from 9022 ILCs for further analysis. Principal component analysis (PCA) dimensionality reduction was performed with the highly variable genes as input. PCs 1–12 were chosen for dimension reduction analysis. We then used the PCs to calculate t-SNE for each dataset. Further analyses including normalization, scaling, clustering of cells, and identifying marker genes were performed by using Seurat (version 3.1.5). Trajectory analysis of ILC2s was performed by using Monocle (version 2.16.0).<br>For flow cytometry data analysis, we used FlowJo (version 10) and numerical data exported to Excel sheet were further analyzed by using GraphPad Prism (version 7) for statistical analysis.

For manuscripts utilizing custom algorithms or software that are central to the research but not yet described in published literature, software must be made available to editors and reviewers. We strongly encourage code deposition in a community repository (e.g. GitHub). See the Nature Portfolio guidelines for submitting code & software for further information.

Data

Policy information about availability of data

All manuscripts must include a data availability statement. This statement should provide the following information, where applicable:

- Accession codes, unique identifiers, or web links for publicly available datasets
- A description of any restrictions on data availability
- For clinical datasets or third party data, please ensure that the statement adheres to our policy

The scRNA sequencing and bulk RNA sequencing data in this publication have been deposited in the National Microbiology Data Center (https://nmdc.cn/resource/genomics/sra) under the accession code NMDC10018099.

Field-specific reporting

Please select the one below that is the best fit for your research. If you are not sure, read the appropriate sections before making your selection.

☒ Life sciences ☐ Behavioural & social sciences ☐ Ecological, evolutionary & environmental sciences

For a reference copy of the document with all sections, see nature.com/documents/nr-reporting-summary-flat.pdf

Life sciences study design

All studies must disclose on these points even when the disclosure is negative.

Sample size	Sample sizes for mouse studies were selected so as to power non-parametric statistical analyses.
Data exclusions	No data were excluded from the analyses.
Replication	As stated in the figure legends, data were either pooled from at least three or four independent replicate experiments; or representative data are shown.
Randomization	Allocation of mice to treatment groups was randomized.
Blinding	No blinding was performed. The samples were prepared, treated and analysed by the same standard procedure. The investigators did not expect the experimental results and most of the test data was automatically generated by the instrument.

Reporting for specific materials, systems and methods

We require information from authors about some types of materials, experimental systems and methods used in many studies. Here, indicate whether each material, system or method listed is relevant to your study. If you are not sure if a list item applies to your research, read the appropriate section before selecting a response.

Materials & experimental systems

n/a	Involved in the study	
☐	☒	Antibodies
☒	☐	Eukaryotic cell lines
☒	☐	Palaeontology and archaeology
☐	☒	Animals and other organisms
☒	☐	Human research participants
☒	☐	Clinical data
☒	☐	Dual use research of concern

Methods

n/a	Involved in the study	
☒	☐	ChIP-seq
☐	☒	Flow cytometry
☒	☐	MRI-based neuroimaging

Antibodies

Antibodies used	Antibodies used for flow cytometry are as follows: anti-mouse CD3-eFluor 450 (Invitrogen, Cat# 48-0032-82, 1:500), anti-mouse CD19-eFluor 450 (Invitrogen, Cat# 48-0193-82, 1:500), anti-mouse CD8a-eFluor 450 (Invitrogen, Cat# 48-0081-82, 1:500), anti-mouse CD11b-eFluor 450 (Invitrogen, Cat# 48-0112-82, 1:500), anti-mouse CD11c-eFluor 450 (Invitrogen, Cat# 48-0114-82, 1:500), anti-mouse Gr1-eFluor 450 (Invitrogen, Cat# 48-5931-82, 1:1000), anti-mouse F4/80-eFluor 450 (Invitrogen, Cat# 48-4801-82, 1:500), anti-mouse Ter119-eFluor 450 (Invitrogen, Cat# 48-5921-82, 1:1000), anti-mouse CD45.2-PE/Cyanine7 (Biolegend, Cat# 109829, 1:1000), anti-mouse CD127-PerCP-eFluor 710 (Invitrogen, Cat# 46-1273-82, 1:300), anti-mouse CD117-PE/Cyanine7 (Invitrogen, Cat# 25-1171-82, 1:500), anti-mouse NK1.1-eFluor 450 (Invitrogen, Cat#48-5941-82, 1:500), , anti-mouse ST2-PE (Invitrogen, Cat# 12-9333-82, 1:500), anti-mouse KLRG1-APC (Invitrogen, Cat# 17-5893-82, 1:500), anti-mouse ROR t-APC (Invitrogen, Cat# 17-6988-82, 1:300), anti-mouse CD45.1-PE/Cyanine7 (Biolegend, Cat# 110715, 1:500), anti-mouse CCR2-PE/Cyanine7 (Biolegend,

Cat# 150611, 1:100), anti-CCR4-PE/Cyanine7 (Biolegend, Cat# 131213, 1:100), and anti-mouse Sca1-FITC (Biolegend, Cat#108105, 1:500).

Validation

All the antibodies used in our study are well-validated antibodies that are commercially readily available.
anti-mouse CD3-eFluor 450 (Invitrogen, Cat# 48-0032-82, 1:500) https://www.thermofisher.cn/cn/zh/antibody/product/CD3-Antibody-clone-17A2-Monoclonal/48-0032-82,
anti-mouse CD19-eFluor 450 (Invitrogen, Cat# 48-0193-82, 1:500) https://www.thermofisher.cn/cn/zh/antibody/product/CD19-Antibody-clone-eBio1D3-1D3-Monoclonal/48-0193-82,
anti-mouse CD8a-eFluor 450 (Invitrogen, Cat# 48-0081-82, 1:500) https://www.thermofisher.cn/cn/zh/antibody/product/CD8a-Antibody-clone-53-6-7-Monoclonal/48-0081-82,
anti-mouse CD11b-eFluor 450 (Invitrogen, Cat# 48-0112-82, 1:500) https://www.thermofisher.cn/cn/zh/antibody/product/CD11b-Antibody-clone-M1-70-Monoclonal/48-0112-82,
anti-mouse CD11c-eFluor 450 (Invitrogen, Cat# 48-0114-82, 1:500) https://www.thermofisher.cn/cn/zh/antibody/product/CD11c-Antibody-clone-N418-Monoclonal/48-0114-82,
anti-mouse Gr1-eFluor 450 (Invitrogen, Cat# 48-5931-82, 1:1000) https://www.thermofisher.cn/cn/zh/antibody/product/Ly-6G-Ly-6C-Antibody-clone-RB6-8C5-Monoclonal/48-5931-82,
anti-mouse F4/80-eFluor 450 (Invitrogen, Cat# 48-4801-82, 1:500) https://www.thermofisher.cn/cn/zh/antibody/product/F4-80-Antibody-clone-BM8-Monoclonal/48-4801-82,
anti-mouse Ter119-eFluor 450 (Invitrogen, Cat# 48-5921-82, 1:1000) https://www.thermofisher.cn/cn/zh/antibody/product/TER-119-Antibody-clone-TER-119-Monoclonal/48-5921-82,
anti-mouse CD45.2-PE/Cyanine7 (Biolegend, Cat# 109829, 1:1000) https://www.biolegend.com/en-us/products/pe-cyanine7-anti-mouse-cd45-2-antibody-4918,
anti-mouse CD127-PerCP-eFluor 710 (Invitrogen, Cat# 46-1273-82, 1:300) https://www.thermofisher.cn/cn/zh/antibody/product/CD127-Antibody-clone-eBioSB-199-SB-199-Monoclonal/46-1273-82,
anti-mouse CD117-PE/Cyanine7 (Invitrogen, Cat# 25-1171-82, 1:500) https://www.thermofisher.cn/cn/zh/antibody/product/CD117-c-Kit-Antibody-clone-2B8-Monoclonal/25-1171-82,
anti-mouse NK1.1-eFluor 450 (Invitrogen, Cat#48-5941-82, 1:500) https://www.thermofisher.cn/cn/zh/antibody/product/NK1-1-Antibody-clone-PK136-Monoclonal/48-5941-82,
anti-mouse ST2-PE (Invitrogen, Cat# 12-9333-82, 1:500) https://www.thermofisher.cn/cn/zh/antibody/product/IL-33R-ST2-Antibody-clone-RMST2-33-Monoclonal/12-9333-82,
anti-mouse KLRG1-APC (Invitrogen, Cat# 17-5893-82, 1:500) https://www.thermofisher.cn/cn/zh/antibody/product/KLRG1-Antibody-clone-2F1-Monoclonal/17-5893-82,
anti-mouse RORt-APC (Invitrogen, Cat# 17-6988-82, 1:300) https://www.thermofisher.cn/cn/zh/antibody/product/ROR-gamma-t-Antibody-clone-AFKJS-9-Monoclonal/17-6988-82,
anti-mouse CD45.1-PE/Cyanine7 (Biolegend, Cat# 110715, 1:500) https://www.biolegend.com/en-us/products/apc-cyanine7-anti-mouse-cd45-1-antibody-2320,
anti-mouse CCR2-PE/Cyanine7 (Biolegend, Cat# 150611, 1:100) https://www.biolegend.com/en-us/products/pe-cyanine7-anti-mouse-cd192-ccr2-antibody-13337,
anti-CCR4-PE/Cyanine7 (Biolegend, Cat# 131213, 1:100) https://www.biolegend.com/en-us/products/pe-cyanine7-anti-mouse-cd194-ccr4-antibody-6333,
anti-mouse Sca1-FITC (Biolegend, Cat#108105, 1:500) https://www.biolegend.com/en-us/products/fitc-anti-mouse-ly-6a-e-sca-1-antibody-227.

Animals and other organisms

Policy information about studies involving animals; ARRIVE guidelines recommended for reporting animal research

Laboratory animals

Ccr2-mNeonGreen-Cre, Cc4-mNeonGreen-Cre, Ccr4-/- and Nr4a1-/- mice were generated by Cyagen Biosciences. Ccr2RFP/RFP and Rosa26-STOP-DTR mice were from Jackson laboratory. Rosa26-STOP-tdTomato mice were from Shanghai Research Center for Model Organisms. B-NDG (NOD-PrkdcscidIL2rgtm1/Bcgen) mice were from Beijing Biocytogen. ILC2 depletion mice Rosa26-STOP-DTR;Ccr2-mNeonGreen-Cre were obtained by crossing Rosa26-STOP-DTR mice with Ccr2-mNeonGreen-Cre mice. Both female and male mice were used in experiments. Age- and sex-matched littermates between 8 and 16 weeks of age were used. Mice were assigned randomly to experimental groups. CD45.1 and B-NDG mice were BALB/c background. All the other mouse strains are C57BL/6 background. Both male and female mice were used. Mice were maintained under specific pathogen-free conditions.

Wild animals

No wild animals were used in this study.

Field-collected samples

No field-collected samples were used in this study.

Ethics oversight

All experimental procedures performed on mice used in this study are in accordance with guideline of Institutional Committee of Institute of Microbiology, Chinese Academy of Sciences.

Note that full information on the approval of the study protocol must also be provided in the manuscript.

Flow Cytometry

Plots

Confirm that:

☒ The axis labels state the marker and fluorochrome used (e.g. CD4-FITC).

☒ The axis scales are clearly visible. Include numbers along axes only for bottom left plot of group (a 'group' is an analysis of identical markers).

☒ All plots are contour plots with outliers or pseudocolor plots.

☒ A numerical value for number of cells or percentage (with statistics) is provided.

Methodology

Sample preparation	For lung ILCs: Lung tissues from mice were cut into pieces and placed in RPMI-1640 containing 2% (vol/vol) FBS (Thermo Fisher Scientific), Collagenase II and III (1 mg/ml; Worthington), DNase I (200 µg/ml; Roche), and incubated for 45 min at 37 ° C. For intestinal ILCs: Intestines of mice were isolated and cleaned. The intestines were cut into pieces after the removing of Peyer's patches. Epithelial layers were removed by incubation three times in 5 mM EDTA Ca2+ and Mg2+ free Hank's medium for 20 min each at 37 °C, and the epithelial cells were collected if needed. Then, intestines were cut into fine pieces and digested twice for 45 min each at 37 °C with RPMI-1640 containing 2% (vol/vol) FBS Collagenase II and III (1 mg/ml; Worthington), DNase I (200 µg/ml; Roche) and dispase (4U/ml; Sigma). All cell suspensions were passed 70 mm cell strainers, and washed twice with PBS to recover cells. Isolated cells were blocked with anti-CD16/32 antibody for 30 min on ice and then stained with antibodies against CD45, CD127, lineage cocktail (Lin=CD3e,CD8a,CD19,CD11b,CD11c,Gr1,F4/80,Ter119) and ILC markers on ice for 1 h followed by 7AAD staining.
Instrument	BD AriaIII for cell purification and analysis.
Software	FlowJo was used to gate the target populations, and Graphpad Prism was used for further analysis.
Cell population abundance	Purity of isolated ILC cells was over 95% for each assay that was determined by post sorting analysis of flow cytometry.
Gating strategy	For all the experiments involving flow cytometry , the following gating strategy was applied to eliminate non-specifically stained cells. First, lymphocyte gate was applied on the SSC-A/FSC-A window. The gating size was determined by comparing the lymphocyte cluster from lymph node samples to the matched tumor samples in order to adjust for the blasted tumor infiltrating lymphocyte cell size. Subsequently, sequential singlet/doublet discrimination was applied via SSCW/SSC-H and FSC-W/FSC-H. From the selected singlets, dead cells were discriminated out by gating on live/dead dye negative population. Gates drawn based on single-stain and full-minus-one (FMO) control.

☒ Tick this box to confirm that a figure exemplifying the gating strategy is provided in the Supplementary Information.
